# Comparative Transcriptomics and Gene Knockout Reveal Virulence Factors of *Neofusicoccum parvum* in Walnut

**DOI:** 10.3389/fmicb.2022.926620

**Published:** 2022-07-15

**Authors:** Jie Chen, Shan Han, Shujiang Li, Ming Wang, Hanmingyue Zhu, Tianmin Qiao, Tiantian Lin, Tianhui Zhu

**Affiliations:** ^1^Department of Forest Protection, College of Forestry, Sichuan Agricultural University, Chengdu, China; ^2^Ecological Institute, Academy of Sichuan Forestry and Grassland Inventory and Planning, Chengdu, China

**Keywords:** *Neofusicoccum parvum*, walnut, gene function, ATP-binding cassette transporter, differentially expressed genes

## Abstract

*Neofusicoccum parvum* can cause stem and branch blight of walnut (*Juglans spp*.), resulting in great economic losses and ecological damage. A total of two strains of *N. parvum* were subjected to RNA-sequencing after being fed on different substrates, sterile water (K1/K2), and walnut (T1/T2), and the function of *ABC1* was verified by gene knockout. There were 1,834, 338, and 878 differentially expressed genes (DEGs) between the K1 vs. K2, T1 vs. K1, and T2 vs. K2 comparison groups, respectively. The expression changes in thirty DEGs were verified by fluorescent quantitative PCR. These thirty DEGs showed the same expression patterns under both RNA-seq and PCR. In addition, *ΔNpABC1* showed weaker virulence due to gene knockout, and the complementary strain *NpABC1c* showed the same virulence as the wild-type strain. Compared to the wild-type and complemented strains, the relative growth of *ΔNpABC1* was significantly decreased when grown with H_2_O_2_, NaCl, Congo red, chloramphenicol, MnSO_4_, and CuSO_4_. The disease index of walnuts infected by the mutants was significantly lower than those infected by the wild-type and complementary strains. This result indicates that ABC1 gene is required for the stress response and virulence of *N. parvum* and may be involved in heavy metal resistance.

## Introduction

Walnuts are a very popular nut and are distributed in temperate to subtropical regions of south America, Asia, Europe, the former Soviet Republics, Oceania, and North Africa (McGranahan and Leslie, 2008). Walnut trees have strong adaptability, cold resistance, and drought resistance and can be planted on flat land, mountains, and hills (Tian et al., 2010). Walnuts can be processed into various foods, including an extracted edible oil. Additionally, because it is rich in polyunsaturated fatty acids, it may have a preventive effect against chronic diseases (Banel and Hu, 2009; Gillingham et al., 2011). However, in the recent years, walnut stem and branch blight caused by *Neofusicoccum parvum* has occurred in several areas of Sichuan, China, resulting in the plant death and large economic losses. *N. parvum* is a destructive wood-infecting fungus distributed all over the world that has a wide host range, high virulence, and strong infection ability. It can infect many important plants and cause fatal branch blight. In addition to walnuts (Kc and Vallad, 2016), *N. parvum* can also infect grapevine (Úrbez-Torres and Gubler, 2011), blueberry (Espinoza et al., 2009), mango (Javier-Alva et al., 2009), kiwifruit (Zhou et al., 2015), avocado (Zea-Bonilla et al., 2007), and olives (Lazzizera et al., 2008).

Blanco-Ulate et al. (2013) first draft the genome of *N. parvum* was assembled using short-read shotgun sequencing and was used to identify a large number of genes encoding cell wall-degrading enzymes (CWDEs), as well as several genes involved in secondary metabolism. Then, based on the whole genome information for *N. parvum*, transcriptome profiling by RNA-sequencing (RNA-seq) showed the genome-wide expression patterns of virulence factors both *in vitro* and in plant. Coexpressed gene clusters were significantly enriched in genes associated with secondary metabolism and cell wall degradation, suggesting that dynamic coregulation of transcriptional networks contributes to multiple aspects of *N. parvum* virulence (Massonnet et al., 2016). The ability to colonize woody tissue and secrete nonspecific phytotoxins is considered to constitute the basis of the pathogenicity and virulence of it (Bruno and Sparapano, 2006; Gómez et al., 2016). Toxins can injure host cells by interfering with enzymatic reactions and cellular transport and damaging cell membranes, and *N. parvum* is able to produce a diverse variety of phytotoxins. Purified hydroxymellein and sphaeropsidone produced *in vitro* by *N. parvum* caused severe foliar symptoms, and terremutin and mellein have been identified in grapevine wood with Botryosphaeria dieback symptoms (Abou-Mansour et al., 2015).

Plant pathogen attacks are perceived through pathogenesis compounds or plant-derived molecules that elicit defense reactions, comprising both constitutive and inducible barriers (Bruno and Sparapano, 2006). Active defense mechanisms mainly involve in the accumulation of phytoalexins, rapid and localized cell death, the synthesis of pathogenesis-related proteins, and reactive oxygen species (ROS) produced in the plant during oxidative burst (Bruno and Sparapano, 2006). Upon infection, plants synthesize many compounds, such as phytoalexins, phenolics, and glycolic acid. At the same time, pathogens produce enzymes to degrade the antifungal compounds released by the host cell to protect themselves, and cell wall-degrading enzymes (CWDEs) are the most abundant and important enzymes due to their roles in penetration, invasion, and pathogenicity. These enzymes can facilitate pathogen invasion and disease development. Therefore, pathogenic fungi require efficient cellular transportation systems to ensure the interaction with host plant and environment, and the ATP-binding cassette (ABC) superfamily proteins are involved in it.

The ABC protein superfamily is one of the largest gene families known to us. They are classified into nine subfamilies from A to I. Its members are reported in every living organisms, ranging from archaea and bacteria to higher eukaryotes (Dassa and Bouige, 2001). But subfamily not yet reported in plants and fungi (Verrier et al., 2008). The canonical structure of the ABC transporters consists of transmembrane domain (TMD) and nucleotide-binding domain (NBD). The TMD consists of the binding site for substrates and is the transport channels of biomembrane. The NBD is the region where ATP is bound and hydrolyzed. Full transporters (FT) possess two sets of TMD and NBD (members of subfamilies A, B, C, and G) whereas the half transporters (HF) possess a single set (members of subfamilies B, D, and G). The ABCE, F, and I subfamily members are soluble proteins consisting of only one or two NBDs and the lack of TMD (Locher, 2009; Kovalchuk and Driessen, 2010; Dube et al., 2018), whereas subfamily I proposed to include “prokaryotic-type” transporters encoded by eukaryotic genomes (Verrier et al., 2008; Kovalchuk and Driessen, 2010). The majority of the ABC proteins are involved in the transport of a broad range of substrate biological membranes, including metal ions, fatty acids, toxins, and other secondary metabolites (Morita and Imanaka, 2012). Notably, several studies have demonstrated the involvement of fungal ABC genes in virulence and fungicide tolerance (Dube et al., 2018). For example, ABC2 from the *Magnaporthe grisea* acts as a multidrug transporters (Lee et al., 2005), whereas *FgABC1* and *FgABC3* of *Fusarium graminearum* are responsible for virulence on wheat, barley, and maize, *FgABC3* and *FgABC4* are involved in triazoles and fenarimol tolerance (Abou Ammar et al., 2013), and *BcatrB* from *Botrytis cinerea* plays an important role in multidrug resistance (MDR) (Vermeulen et al., 2001).

When pathogens interact with host plants, the gene expression of the pathogen will change with the metabolic changes of the host plant. To understand the change, RNA-seq has been used to analyze the metabolic changes and virulence factors involved in pathogen infection (Gao et al., 2011; Wurtzel et al., 2012; Yan et al., 2012; Inglis et al., 2013; De Fine Licht et al., 2016), including genomic clusters of virulence factor analysis about the process of *N. parvum*-infecting grapevine. However, the active pathogenic substances produced by the same fungus in different hosts and under different conditions can vary (Li, 2012; Li et al., 2013). It is necessary to screen the genes related to *N. parvum* infection and pathogenicity in other plant hosts by transcriptome analysis, as the functions of these candidate genes during infection are unclear. Gene knockout is an important means to study gene function. In the recent years, gene knockout methods for filamentous fungi have become increasingly mature (Colot et al., 2006; Al Abdallah et al., 2017). Nowadays, the functions of several genes in many important plant pathogenic fungi, such as *Magnaporthe grisea* (Wu et al., 2006), *Botrytis cinerea* (Roca et al., 2012), and *Colletotrichum gloeosporioides* (Yakoby et al., 2001), have been confirmed. We sequenced and compared the transcriptomes of two strains of *N. parvum in vitro*. The genes with significant differences were verified by quantitative real-time PCR. Besides, we explored gene function by gene knockout. In this study, we provide reliable reference data for further study on the pathogenic mechanism of *N. parvum* and explore the function of the ATP-binding cassette transporter in *N. parvum* pathogenicity.

## Materials and Methods

### Materials

#### Microorganism and Plant

*N. parvum* was isolated by the tissue isolation method (Koonin et al., 2004) from branch blight disease of walnut. The accession numbers of the ITS, tef1, and TUB2 genes for strain BH01 in the NCBI database are OM980641, ON000394, and ON000396, the accession numbers of the ITS, tef1, and TUB2 genes for strain BH03 in the NCBI database are OM980642, ON000395, and ON000397, respectively. The plant tissue samples were collected from 5-year-old walnut branch blight samples in Ya'an city (102°59′N, 29°58′E), altitude: 738.35 m, annual temperature: 6.1–25.3°C, and annual precipitation: 1,204–2,367 mm. The pathogenicity test plants were purchased from Sichuan Chengdu Fujiao Seeding Co., Ltd., China and planted in the greenhouse of Sichuan Agricultural University in Chengdu city (30°42′ N, 103°51′ E).

### Methods

#### Biological Material

The method of Andrade et al. (2008) was used to obtain sterile walnut plant tissue extractions and stored it at −20°C. Then, we combined the method of Andrade et al. (2008) with the method of Morales-Cruz et al. (2015), to perform transcriptome profiling of fungal cultures on solid medium. A total of two strains of *N. parvum* were inoculated onto the potato dextrose agar (PDA) coated with 2 ml of sterile water to collect K1-1, K1-2, and K1-3 (three biological replications of strain BH01) and K2-1, K2-2, and K2-3 (three biological replications of strain BH03). At the same time, two strains were inoculated on the SuperPure agar containing fine ground walnut wood coated with 2 ml sterile walnut extract to collect T1-1, T1-2, and T1-3 (three biological replications of strain BH01) and T2-1, T2-2, and T2-3 (three biological replications of strain BH03). Cultures were incubated at 25°C for 7 days, subcultured into the same medium, and incubated again for 7 days. Subculturing was carried out three times before RNA extraction of mycelium harvested from the surface of the agar.

#### RNA Extraction, Library Preparation, and Sequencing

Mycelium from cultures grown on solid medium was collected in a 2-ml microcentrifuge tube, immediately frozen in liquid nitrogen, and then ground to a powder. Then, total RNAs were extracted following the manufacturer's protocol of TransZol Up (transgenbiotech, China). Then, the genomic DNA was removed by DNase, RNase inhibitor (TakaRa, China), and other reagents. Next, 1% agarose gel electrophoresis, a NanoPhotometer spectrophotometer (IMPLEN, CA, USA), and a Bioanalyzer 2100 system (Agilent Technologies, CA, USA) were used to test the total RNA quality, purity, and integrity, respectively. RNA-seq libraries were prepared using the NEBNext^®^ Ultra RNA Library Prep Kit for Illumina (NEB, USA) following the manufacturer's recommendations, and index codes were added to attribute sequences to each sample. Finally, the PCR products were purified (AMPure XP system), and library quality was assessed on the Agilent Bioanalyzer 2100 system. The clustering of the index-coded samples was performed on a cBot Cluster Generation System using TruSeq PE Cluster Kit v3-cBot-HS (Illumina) according to the manufacturer's instructions. After cluster generation, the library preparations were sequenced on an Illumina HiSeq4000 sequencer platform by Novogene Co., Ltd. (Beijing, China), and 150 bp paired-end reads were generated.

#### Quality Control, Reference Genome, and Novel Transcript Prediction

Raw data (raw reads) in fastq format were first processed through in-house Perl scripts. In this step, clean data (clean reads) were obtained by removing reads containing adapters, reads containing poly-N sequences, and low-quality reads from the raw data. At the same time, the Q20, Q30, and GC contents of the clean data were calculated. All downstream analyses were based on clean, high-quality data. Sequencing fragments (reads) are randomly interrupted by mRNA. To determine which genes are transcribed, we need to compare the clean reads after quality control to the reference genome. The reference genome and annotation files were downloaded from publicly available data submitted to NCBI by Blanco-Ulate et al. (2013). The index of the reference genome was built using HISAT2 v2.0.5 (Mortazavi et al., 2008). The mapped reads of each sample were assembled by StringTie v1.3.3b(Pertea et al., 2015) *via* a reference-based approach.

#### Quantification of Gene Expression Levels and Analysis of DEGs

Feature Countsv 1.5.0-p3 (Liao et al., 2014) was used to obtain the number of reads covering each gene from start to finish according to the location information based on the reference genome (the reads with alignment quality values lower than 10, the reads mapped to non-comparative pairs, and the reads from multiple regions of the genome were filtered out). Then, the expression values were calculated based on the expected number of fragments per kilobase of transcript sequence per million base pairs sequenced (FPKM) (Trapnell et al., 2012) for all genes in each sample. The sequencing depth and gene length were corrected (Bray et al., 2015). The differential expression of two strains of pathogenic fungi in sterile water on PDA and sterile hybrid walnut tissue extract on walnut wood pure agar was determined using the DESeq2 R package (1.16.1) (Love et al., 2014). First, the original read count was normalized, mainly to correct the sequencing depth. Then, the statistical model calculated the hypothesis test probability (*p*-value) and performed multiple hypothesis test corrections to obtain the padj value (Anders and Huber, 2010). Genes with significant differences in expression were defined as having a false discovery rate |log2(FoldChange)| > 2 and padj □ 0.05. Gene ontology (GO) (Kanehisa et al., 2004) functional analysis and KEGG (Koonin et al., 2004) pathway (http://www.genome.jp/kegg/) analysis of DEGs were carried out to search for virulence genes. The methods of GO enrichment and KEGG pathway analysis of DEGs according to Wang et al. (2022).

#### Verification by Quantitative Real-Time PCR (qRT-PCR)

A total of ten DEGs were randomly selected in each of the three comparison groups. Finally, a total of 30 DEGs were screened. Primer3Plus (http://primer3plus.com/cgi-bin/dev/primer3plus.cgi) was used to design primers for 30 candidate genes and reference gene GAPDH (Table 1). The CFX96 Real-Time PCR Detection System (BIO-AD, USA) was used to quantify the changes in the expression of thirty genes across all twelve samples. First, cDNA was obtained using the PrimeScript RT Reagent Kit with gDNA Eraser (TaKaRa) following the manufacturer's recommendations. The qPCR system was performed using TB Green^®^ Fast qPCR Mix (TaKaRa) as follows (20 μl total): 10 μl of TB Green Fast qPCR Mix, 6.4 μl of ddH_2_O, 0.8 μl of forward and reverse primers, and 2 μl of cDNA (10-fold dilution). The qPCR conditions were as follows: 95°C for 30 s, followed by 40 cycles of 95°C for 5 s, and 60°C for 10 s, and a melting curve. Each reaction was repeated three times, and the average value was calculated. GAPDH was used as an internal reference gene to detect the expression changes in the different treatment groups. Data were analyzed by the 2^−ΔΔCt^ method (Livak and Schmittgen, 2001).

**Table 1:** The primer sequences of DEGs and reference gene for qRT-PCR.

**Gene type**	**Gene**	**Log2FC**	**Gene description**	**Primer sequence 5^**′**^-3^**′**^(F;R)**
Targets gene K1 vs. K2	*SDR*	11.6448	Short-chain dehydrogenase reductase sdr protein	TTCACGCGCATTATGCAGTC; GCTCTTCACCTTGAACAacgac
	*STF*	9.4561	Fungal specific transcription factor domain-containing protein	ACTACATGTTCGACGACAGC; AGTCTTTGGCGTTGATCTCg
	*STP*	6.3147	Sugar transporter protein	AGAAATGTGCCGCTTTCAGC; AGGTGCCGAATTGCTTTTCG
	*p450*	5.2998	Cytochrome p450 protein	GCGCAACCTGTTTTTCATGG; GCCATCatccttctgcttcttg
	*NmrAL*	6.8858	NmrA-like	TGCAAAAGAGTGGAGCAAGG; TTCCAGTTGGTTGCGTACAG
	*ABC1*	4.2364	abc transporter protein	TGCAACAATATCGCGGCATC; ACATGACATCGTTGGCCTTG
	*MFS*	5.2763	mfs transporter protein	AAGCAGCATTTACGGCAGTG; tgaTGCCAAGAGTTGTCACG
	*PTH11*	−3.6987	Integral membrane protein pth11 protein	ACCTCACCAACTCCATCATCAG; CACGACGCTGAGTTTGATCTTG
	*DJ-1*	−7.0738	dj-1 family protein	TTGTACACCATGGCTTTCGG; GGGTCGAATTGGTGTGTCTTC
	*OxDC*	−7.9939	Oxalate decarboxylase protein	TGCTGCATCGAGGACTTTTG; AAGCGAGATATCAGCAACGC
Targets gene T1 vs. K1	*mbl*	−7.7325	Metallo-beta-lactamase	ATGCGAGGGAGATTTTTGCG; TTGGTGATGGCCTTTGCAAG
	*MFS_1*	−6.1696	mfs transporter protein	AACCGGTGTTTGGAAAAGCC; ACGAATTCGCCAATGCACAG
	*ELO*	−4.3438	Fatty acid elongase protein	ACCTCTTCGACAAGTCATGGAC; ACGTAGTAGACCGCCAAcatg
	*NOX*	−3.8764	Nadh:flavin oxidoreductase nadh oxidase protein	ACGATGTTGTGGCTGTGTTC; TGGTACACGCTTTCCATCAC
	*SDH*	3.5028	Sorbitol dehydrogenase protein	TTCGCTCATGCTGGATTCAC; ATTCGAACACCGCATcaacc
	*SCP*	6.3604	Allergen v5 tpx-1-related protein	AGTGGGTCAAGTTGCACAAC; CGCCAAGGTTGTTGTTTTGG
	*Gyk*	3.2623	Glycerol kinase protein	AGGGCACAATGTTTGGCATC; TTCGTTTGGTAGCAGGTTGC
	*GH31*	11.5054	Glycoside hydrolase family 31 protein	AGCTACAACCTGCACAAAGC; atgATGAAGTTGCGCTTGCC
	*AQP3*	6.3363	Aquaporin 3 protein	TTGCTCTCAAAGACGACGAC; AGCAAGCACCGATTCCAAAG
	*BBR*	5.0992	Berberine family protein	TCCTTCACGAAACGCTTGTG; TGGCTTCACGAATGATTGCG
Targets gene T2 vs. K2	*p-ATPase*	6.5861	Potassium sodium efflux p-type atpase protein	TTTCCAGCATTCCGTCGAAC; TGGCCTTTTTGGAGCACAAG
	*M35*	5.8362	Peptidase m35 deuterolysin protein	AAGTTCATCGGCAGCCTTTC; TGTTGGTGATCGAAGCCTTG
	*MFS*	4.8503	Major facilitator superfamily domain general substrate transporter protein	TTACATCGTGGGCATCATGC; AAGACCAACACGCTCAAACG
	*pmr1*	7.1314	calcium-Translocating p-type atpase protein	TGTTCAACTGCTTCGTGTGG; TGGCGATGAAGAACCAGTTG
	*MP1*	3.4157	Metalloprotease 1 protein	ATACGTCACGGACCAAATGC; TTCTTCATTGCCAGCTCAGC
	*CBP*	3.5283	Cellulose-binding protein	ACTGTGACGAAACGAGATCCTC; TGATGTGCGCCGTAATGTTG
	*nor-1*	−3.0509	Norsolorinic acid reductase protein	TTGTCCTGGCAACCAAGTTC; TGCTTCAAGCTCTTGGCATG
	*PKSs*	−4.0662	Polyketide synthase protein	ACAAATGCGTCACCCTGAAC; TGGTCGGCTTGTTAAACACC
	*2og-fe*	−5.1855	2og-fe oxygenase family protein	CAACGTCGACATCTCAATGCC; TGTGCGTCTTGAATGTGTGC
	*mtlD*	−7.2224	Mannitol 1-phosphate dehydrogenase protein	TGCGCGAATGCATTCTAAGC; TGACGTTTTCCGCTGCATTG
Reference gene	*GAPDH*	-	Glyceraldehyde-3-phosphate dehydrogenase	CTACTACATCGTCGAGTCCACC; GTGTAGCTCTCGTTGTTGACAC

#### Cloning of ABC1

Primers were designed to obtain ABC1 fragments from gDNA and cDNA. The pClone007 Versatile Simple Plasmid Kit (TSINGKE, China) was used to ligate the fragments. The recombinant plasmid was transformed into *Escherichia coli* strain DH5α and detected by colony PCR.

#### Knockout Plasmid Construction

The KanMx gene was amplified from PUG6 plasmid DNA as a screening marker gene. Moreover, we used an improved split-marker method to identify gene function (Goswami, 2012). Taking the CDS region of the ATP-binding cassette transporter 1 (ABC1) gene as the center, 1,100–1,500 bp regions upstream and downstream were selected as homologous arms to design primers (the complementary sequence of the upstream primer of the KanMx gene was added to the 5' end of the downstream primer of the upstream homologous arm; the complementary sequence of the downstream primer of the KanMx gene was added to the 5′ end of the upstream primer of the downstream homologous arm, as shown in Table 2) to amplify homologous arms of ABC1 from *N. parvum* DNA. The gDNA of *N. parvum* was extracted by Column Fungal DNAout 2.0 (TIANDZ, China). Amplification system: 1 μl of DNA, 1 μl of the forward and reverse primers, 25 μl of 2×TransTaq HiFi PCR SuperMix (TaKaRa), and 22 μl of ddH_2_O. The reaction procedure was as follows: 94°C for 5 min, followed by 35 cycles of (94°C for 30 s, 55°C for 30 s, and 72°C for 1 min 30 s, and a final step of 72°C for 8 min). Upstream/downstream and KanMx gene fragments were obtained. The fusion fragments were obtained by overlap extension PCR (Horton et al., 1989). The amplification system (30 μl total) contained 6 μl of upstream/downstream DNA, 6 μl of KanMx DNA, 3 μl of 10×LA buffer, 1 μl of dNTP Mix, 0.3 μl of LA Taq (TaKaRa), and 13.7 μl of ddH_2_O. The reaction procedure was as follows: 94°C for 5 min, followed by 35 cycles of 94°C for 30 s, 55°C for 1 min, and 72°C for 2 min 30 s, and a final step of 72°C for 8 min. The fusion fragment upstream-KanMx and KanMx-downstream fragments were obtained. The vector pClone007 was used to ligate the fusion fragment. The recombinant plasmid was transformed into DH5α and detected by colony PCR.

**Table 2:** ABC1 Gene knockout and complement experiment primers.

**Primer name**	**Primer sequence 5^**′**^-3^**′**^**	**Purpose**
*ABC1-up-F*	CAAGCCTATTCTGCCTAAC	Amplified homologous upstream
*ABC1-up-R*	*CTGACAAAAGTCCGTCTTTTGGGGCCAT*GCTGTATTGAGACGAGCC	
*ABC1-down-F*	*CAGATCCACTAGTGGCCTATGC*ACGATAGACGAATTTTCCTAA	Amplified homologous downstream
*ABC1-down-R*	GGTGCATCCGGTTACTGC	
KanMX-F	CAGCTGAAGCTTCGTACGC	Amplified resistance KanMx gene
KanMX-R	GCATAGGCCACTAGTGGATCTG	
*ABC1-cF*	tagatgccgaccgcgggatccATGCAGTTTTGGCTACTGGCG	Amplification of ABC1 (from cDNA); Detection of ABC1 gene from deletion mutant strains
*ABC1-cR*	tcagtaacgttaagtggatccTTAATGGCTTTCCGCGCC	
*ABC1-F*	tagatgccgaccgcgggatccTCGCTCCGAAAAGTAGCCG	Amplification of ABC1 (form gDNA); Detection of ABC1 gene from complementary strains
*ABC1-R*	tcagtaacgttaagtggatccCATCCTTCGGTCTTTGGAAGG	
*Detection fragment 1-F*	CTGGCGATGTTGTTCTCCCT	Detection of fragment 1 from deletion mutant strains
*Detection fragment 1-R*	AAACTGGACGGGTATGCTGG	
*Detection fragment 2-F*	CGAAGGCCCTCATCATCCTC	Detection of fragment 2 from deletion mutant strains
*Detection fragment 2-R*	CGTCAATCTTGATGCTGCCG	

*The underlined sequence is the homologous arm sequence of KanMx; The sequence of lowercase letters is the homologous arm sequence of the cleavage site of vector pAN7-1*.

#### Complementation Test ABC of Knockout

The primer pairs ABC1-F(p-up)/ABC1-R(p-down) (Table 2) were designed based on the sequences of the ABC1 gene and the vector pAN7-1 (hygromycin resistance), as well as on the principle of homologous recombination. ABC1 gene fragments were amplified, purified, and recovered. The vector pAN7-1 was digested with the restriction enzyme BamH I, and the fragments were recovered and transformed by homologous recombination with the ABC1 gene fragment to obtain the gene complementing plasmid pAN7-1-ABC1. Restriction digestion was performed with the restriction enzyme BamH I for verification.

#### Protoplast Preparation and PEG-Mediated Protoplast Transformation

The method of protoplasmic preparation of *N. parvum* and PEG-mediated genetic transformation were the same as (Fang et al., 2021). Finally, direct PCR lysis buffer for microorganisms (TaKaRa) was used for transformant detection (refer Table 2 for the detection primers).

#### Phenotypic Analysis and Determination of Sensitivity

To observe the colony morphology of strains and to analyze whether the difference in vegetative growth between the strains, we inoculated the strains on PDA plates (9 × 9 cm) and measured and observed the strains after 3 and 5 days. To determine whether ABC1 plays a role in stress sensitivity, we tested the effects of Congo red (CR), NaCl, and H_2_O_2_ on wild-type and mutant strains. Moreover, to determine whether ABC1 plays a role in multidrug resistance, we tested the effects of antifungal drugs on wild-type and mutant strains. Growth assays on agar plates, EC_50_, and MIC sufficient to suppress fungal mycelial growth were determined (Urban et al., 1999). The tested drugs included protein synthesis inhibitors (chloramphenicol) and sterol biosynthesis inhibitors (carbendazim and tebuconazole). For all substances, stock solutions were prepared in DMSO. To assess transition metals, we tested the effects of metal salts (CuSO_4_ and MnSO_4_) on wild-type and mutant strains. Mycelial plugs with unamended PDA were used as the inoculum to determine the effect of the compounds on vegetative growth. At least three plates were used for each compound and concentration.

#### Pathogenicity Test

The 3-year-old, uniformly grown and healthy *J. regia* were used for the pathogenicity test, and from them, each group of ten plants was selected randomly. Branches with uniform growth from the top three branches of each plant were selected and covered with mycelial plugs of the wild-type strain, mutant Δ*NpABC*1, complementary strain *NpABC1c*, and pure PDA. The samples were subjected to bagging to retain moisture and sprayed with sterile water one time every 24 h. The disease incidence was investigated 20 days after inoculation. The disease severity was assessed regularly using a 0–5 scale: 0 = no infection, 1 = 1–20% infection, 2 = 21–40% infection, 3 = 41–60% infection, 4 = 61–80% infection, and 5 = more than 80% infection (Suharban et al., 2020). The percent disease index (PDI) was determined following the standard formula described by Islam et al. (2012):


(1) $$\begin{array}{r} PDI=\frac{\sum \left(Ni\times Di\right)}{N\times Dm}\times 100 \end{array}$$

Note: D_i_: disease grade, N_i_: number of diseased branches, N: total number of branches, and D_m_: the most serious disease grade.

## Results

### Transcriptome Sequencing and Analysis

#### Quality Control and Read Mapping to the Reference Genome

The Q30 base percentage in each sample after filtering was not <94.90%. The GC content was more than 57.09%. The read lengths of the 12 samples were 150 bp. The transcriptome sequencing results met the quality requirements for subsequent assembly analysis. The sequencing information of the 12 samples is shown in Table 3. All 12 samples generated sequencing reads that aligned to the genome with total mapped reads no <91.84%. Of these, 91.71 to 95.98% of the reads aligned to unique positions of the reference genome, and 0.13 to 0.21% aligned to multiple positions of the reference genome. Statistical results of comparisons between samples and the reference genome are shown in Table 3. We used StringTie v1.3.3b as a reference-based approach to assemble the mapped reads of each sample. Finally, we assembled 2,401 transcripts not annotated to the genome and attempted to annotate them in other databases; among them, 507 were annotated to GO, 85 were annotated to KEGG, and 198 were annotated to Pfam. All transcriptome data of the 12 samples of *N. parvum* were deposited in the NCBI Sequence Reads Archive (SRA) under the accession numbers SRX11046476-11046487.

**Table 3:** The sequencing information of samples and statistics of comparison between sample and reference genome.

**Sample**	**Clean reads**	**Q20**	**Q30**	**GC pct**	**Total map (percent)**	**Unique map**	**Multi map**
K1-1	42144236	98.38	95.24	58.46	39938256 (94.77%)	39859811 (94.58%)	78445 (0.19%)
K1-2	39878812	98.39	95.29	58.33	37542793 (94.14%)	37467025 (93.95%)	75768 (0.19%)
K1-3	42651806	98.4	95.31	58.41	40343592 (94.59%)	40263643 (94.4%)	79949 (0.19%)
T1-1	42701610	98.21	94.9	57.68	39317348 (92.07%)	39258162 (91.94%)	59186 (0.14%)
T1-2	40913706	98.46	95.45	58.32	38673866 (94.53%)	38602955 (94.35%)	70911 (0.17%)
T1-3	40863508	98.21	94.79	57.09	37527141 (91.84%)	37474115 (91.71%)	53026 (0.13%)
K2-1	45608152	98.26	95.02	57.83	43366246 (95.08%)	43269025 (94.87%)	97221 (0.21%)
K2-2	39222022	98.34	95.26	58.21	37544953 (95.72%)	37468838 (95.53%)	76115 (0.19%)
K2-3	44310068	98.41	95.43	58.84	42576880 (96.09%)	42485152 (95.88%)	91728 (0.21%)
T2-1	43929474	98.36	95.26	59.62	42233084 (96.14%)	42163937 (95.98%)	69147 (0.16%)
T2-2	45123124	98.23	94.94	59.12	43265019 (95.88%)	43181708 (95.7%)	83311 (0.18%)
T2-3	42039814	98.55	95.67	59.15	39958704 (95.05%)	39889067 (94.88%)	69637 (0.17%)

#### Quantification of Gene Expression

**Figure 1:**
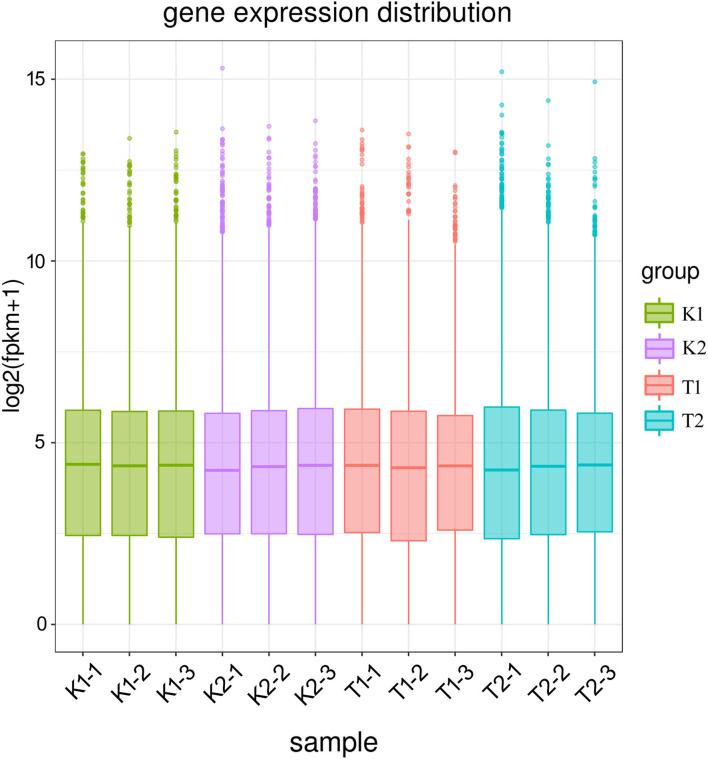
Box plot of gene expression across samples. A total of 12 samples were assigned to four groups; K1 (K1-1, K1-2, K1-3) was the control group of strain BH01; K2 (K2-1, K2-2, K2-3) was the control group of strain BH03; T1 (T1-1, T1-2, T1-3) was the treatment group of strain BH01; Group T2 (T2-1, T2-2, T2-3) was the treatment group of strain BH03. FPKM: expected number of fragments per kilobase of transcript sequence per millions base pairs sequenced.

**Figure 2:**
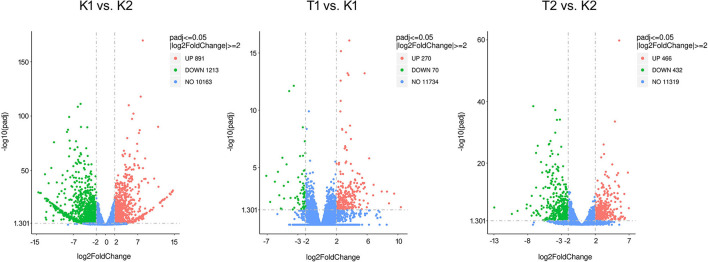
Volcano plot of DEGs in compared groups. The criteria to filter DEGs between compared groups are *p* ≤ 0.05 and |log2FoldChange|≥ 2. The X-axis (log2FoldChange) indicates the fold change in expression of the gene in the compared groups, and the Y-axis (–log10padj) indicates the significance level of the difference in expression of the gene in the compared groups. Red dots indicate upregulated genes and green dots indicate downregulated genes.

The distribution of gene expression levels in different samples is shown in Figure 1. When comparing the DEGs under different pairs of strains and culture conditions, 891 DEGs were found to be upregulated and 1,213 were downregulated in the K1 vs. K2 comparison, 270 genes were upregulated and 72 were downregulated in T1 vs. K1, and 466 genes were upregulated and 433 were downregulated in K2 vs. T2. The numbers of DEGs were similar between the T1 vs. K1 and T2 vs. K2 comparisons, and the number of DEGs in the K1 vs. K2 comparison was large. The results showed significant differences in gene expression patterns between the two strains and a significant difference between those patterns based on the culture conditions of the two strains, either in the walnut tissue medium and in sterile water. The DEGs between different groups under the three comparisons are shown in a volcano plot (Figure 2).

#### GO and KEGG Annotation Analysis of DEGs

**Figure 3:**
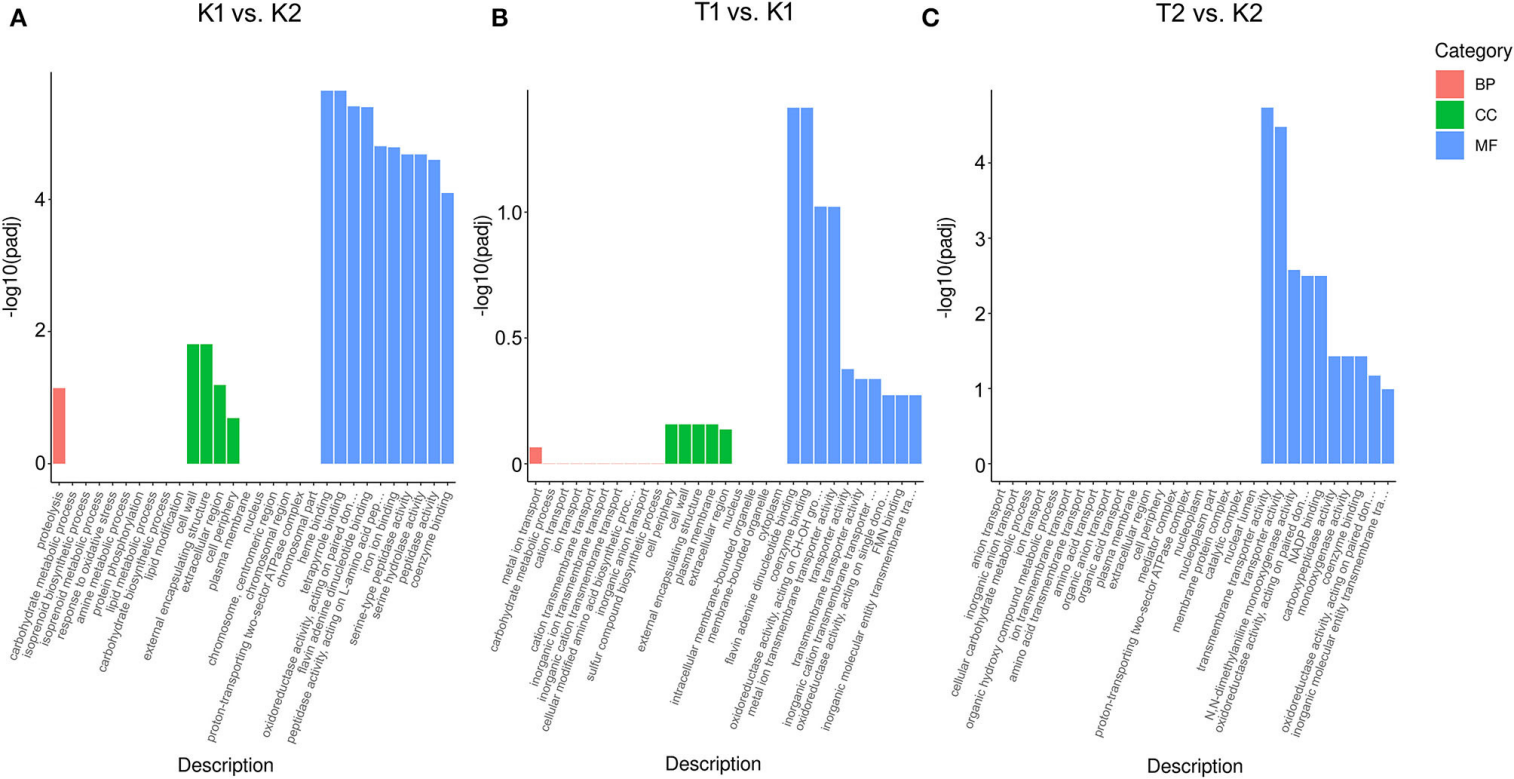
Histogram of GO enrichment analysis of DEGs in compared groups of **(A)** K1 vs. K2, **(B)** T1 vs. K1 and **(C)** T2 vs. K2. The three parts of the GO enrichment result in each compared group exhibited the top 10 terms each, with red columns indicating the biological process, green columns denoting the cellular component, and blue columns indicating the molecular function. Y-axis (–log10padj) indicates the significance difference of GO term enrichment.

Gene ontology functional enrichment analysis was carried out for the DEGs of the two strains of *N. parvum* under different treatment conditions. In the GO enrichment analysis of the K1 vs. K2 comparison group, molecular functions (MF), biological processes (BP), and cell components (CC) were annotated. However, the DEGs were mainly enriched in MF, followed by CC, and a few were enriched in BP (Figure 3A). Among them, the following functions were significantly enriched in MF: redox reaction, peptidase, glycosyl bond hydrolase activity, carbon oxygen lyase activity, iron, copper, zinc ion pump, peroxidase activity, protein kinase activity, signal receptor activity, molecular sensor activity, and transmembrane transporter activity. While protein hydrolysis, carbohydrate anabolism, terpene anabolism, response to oxidative stress, amine metabolism, anion transport, phosphorylation, and signal transduction system were enriched in BP and CC, extracellular activity, plasma membrane, chromosome, membrane protein, and organelle were significantly enriched in CC. In the GO enrichment analysis of the T1 vs. K1 comparison group, the DEGs were mainly enriched in MF, followed by CC, and a few were enriched in BP (Figure 3B). Among them, the following functions were significantly enriched in MF: antioxidant activity, oxidoreductase-related reactions, transmembrane transporter activity of metal ions, glycosyl bonds, carbon nitrogen bonds, ester bonds, amino acids, anhydrides and other hydrolase activities, lyase activity, drug binding, and ATPase activity. The BP category was more enriched in ion transport, carbohydrate metabolism, sulfur and nitride metabolism, small molecule catabolism, response to oxidative stress, protein hydrolysis, nucleic acid transcription and translation, and signal transduction. While the CC category was enriched in plasma membrane, extracellular assembly, cell wall, and cytoplasm, in the GO enrichment analysis of the T2 vs. K2 comparison group, the DEGs were mainly enriched in MF (Figure 3C). The following functions were significantly enriched in MF: transmembrane transporters, oxidoreductases, signal receptors, signal sensors, molecular receptors, peptidases, various lyases, hydrolases, and transferases. BP was mainly enriched in species transmembrane transport, carbohydrate metabolism, response to oxidative stress, and protein hydrolysis, which meant that the metabolic activity of cells was enhanced. CC was partially enriched in some processes related to plasma membrane and membrane protein complex.

**Figure 4:**
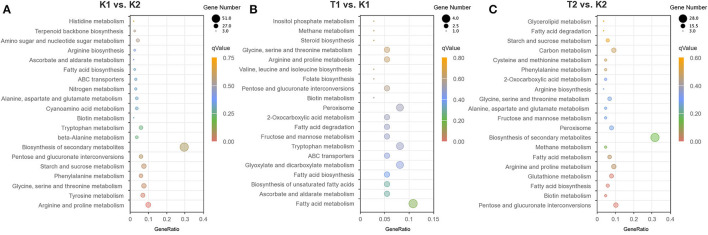
Bubble chart of KEGG enrichment analysis DEGs in compared groups of **(A)** K1 vs. K2, **(B)** T1 vs. K1 and **(C)** T2 vs. K2. The most significant 20 pathway in the KEGG enrichment results from compared groups. The X-axis (GeneRatio) indicates the ratio of the number of genes annotated into that KEGG pathway and the total number in differential genes in that group. The q value indicates the significant difference of KEGG enrichment, indicated by different colors in the plots, and the size of the bubble indicates the number of genes in the pathway.

In the KEGG enrichment analysis of the K1 vs. K2 comparison groups (Figure 4A), 78 enriched pathways were successfully identified, including carbon, nitrogen and sulfur, the metabolism of amino acids, the biosynthesis of secondary metabolites and biotin (participating in a series of important metabolic processes, including sugar, protein, and fatty acid metabolism), ABC transporter, biosynthesis of terpene skeletons, and peroxisome. In the KEGG enrichment analysis of the T1 vs. K1 comparison groups (Figure 4B), 37 enriched pathways were successfully identified, including fatty acid, aldehyde acid, carboxylic acid, folate metabolism, ABC transporter, peroxisome, biotin metabolism, inositol phosphate metabolism, and steroid biosynthesis. In the KEGG enrichment analysis of the T2 vs. K2 comparison groups (Figure 4C), enriched pathways were successfully identified, including amino acid metabolism, biotin metabolism, fatty acid metabolism, secondary metabolite synthesis, peroxisome, carbon metabolism, and ABC transporter (Supplementary Figure S8). Among the DEGs searched, the upregulated expression of the ABC transporter in all comparison groups (the top 30 pathways) may be related to the production of several transporter proteins by *N. parvum* to expel the protective substances and toxins produced by walnut out of the cell. In this case, the ABC gene is considered to be related to *N. parvum* virulence.

### Verification by qRT-PCR

**Figure 5:**
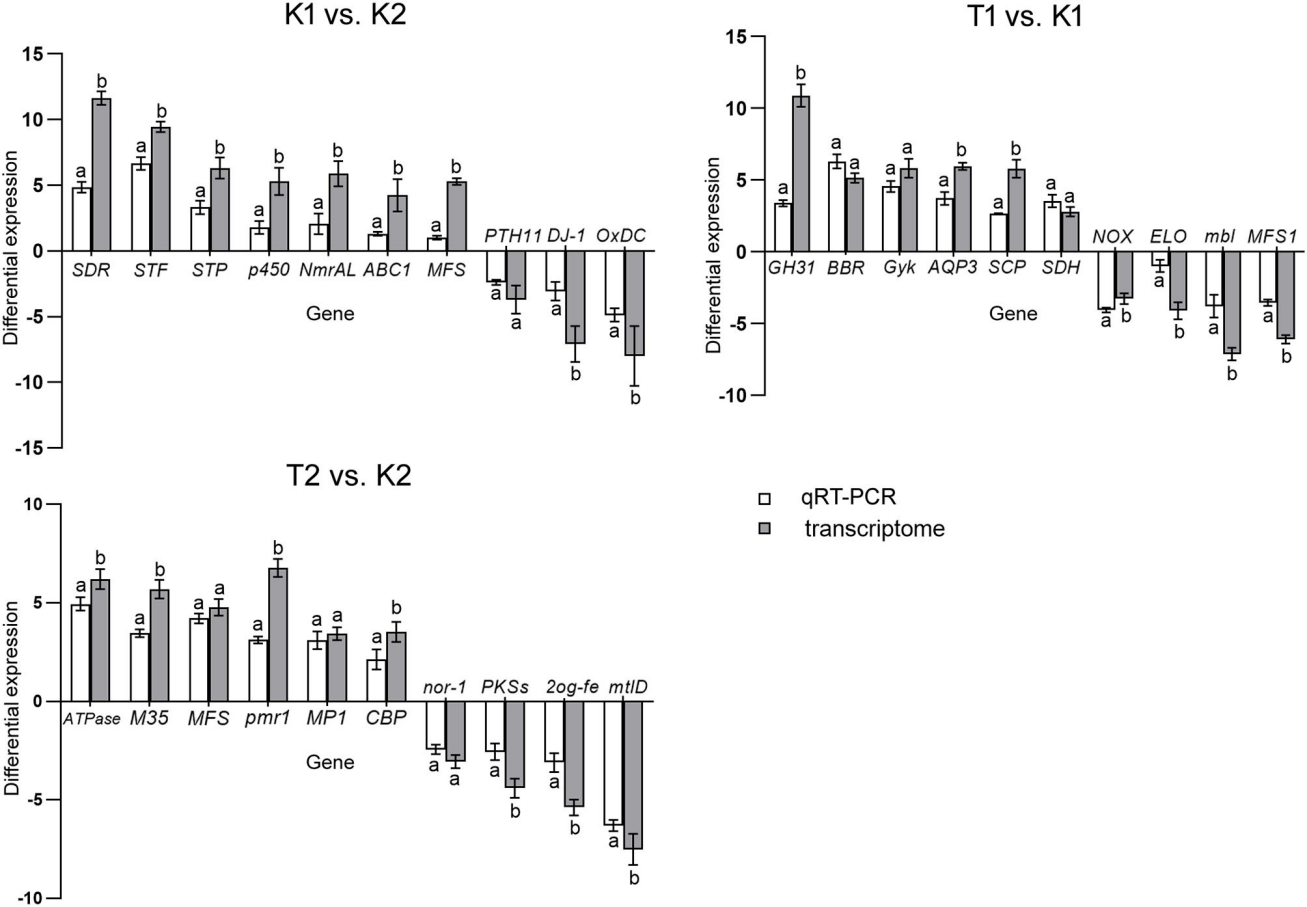
Histogram of relative expression of DEGs by qRT-PCR and transcriptome analysis. All assays were repeated three times, the data were analyzed using one-way ANOVA and Duncan's range test in SPSS 16.0. Different lowercase letters showed that there were significant differences in the expression level of the same gene between the qRT-PCR and transcriptome analysis (*p* ≤ 0.01). White column represents the differential relative expression after normalization with GAPDH as the reference gene in the compared groups. Gray column represents the gene differential expression in the transcriptome differential analysis of the comparison groups.

To verify the reliability of the transcriptome data, qRT-PCR was used to quantify the gene expression levels of the two strains under different conditions. We selected 30 DEGs in total, with 10 DEGs in each comparison group. The results showed a similar trend for the qRT-PCR and transcriptome data (Figure 5).

### Gene Function Verification

#### Cloning of ABC1

**Figure 6:**
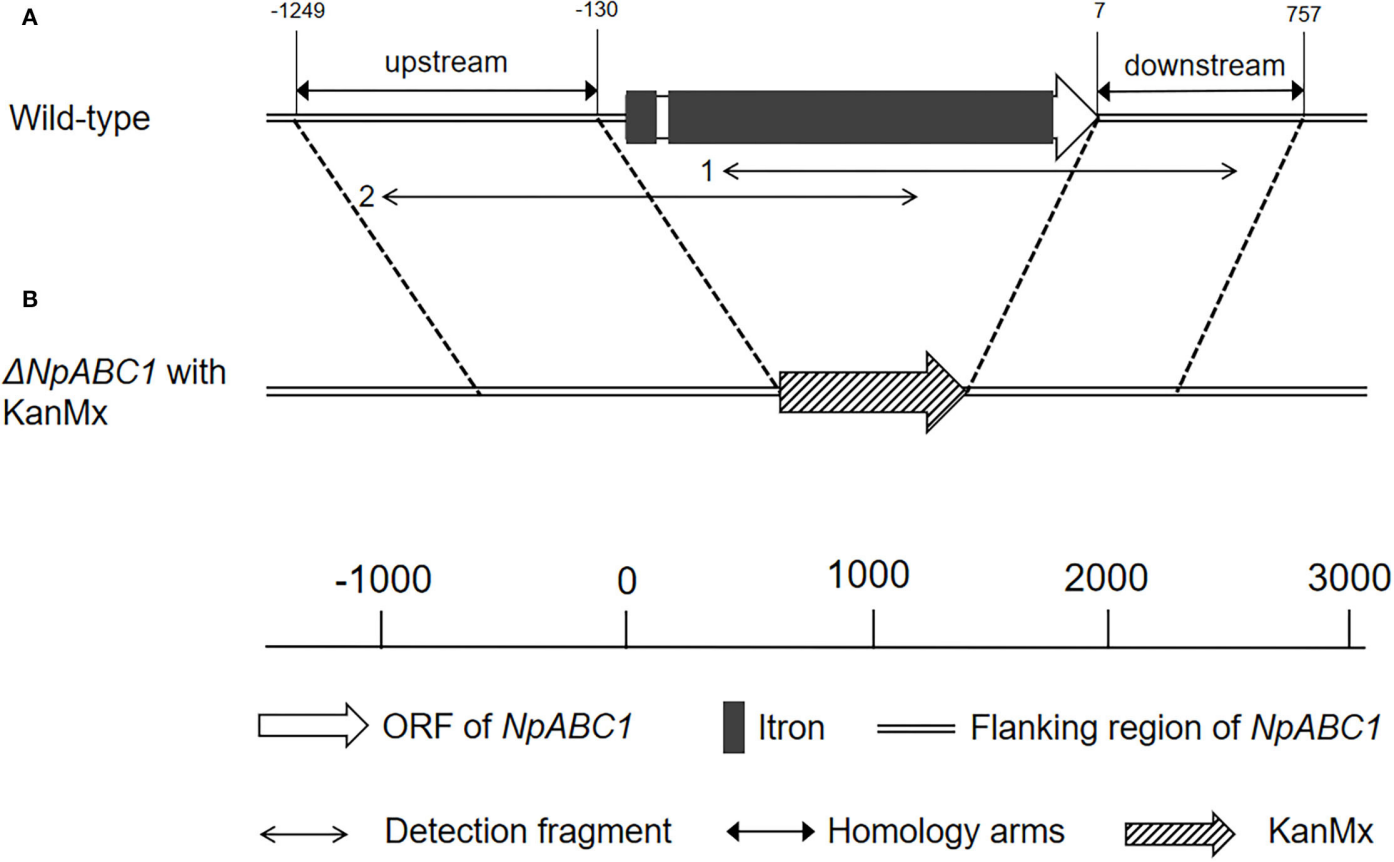
Schematic of ABC1 gene structure and knockout. **(A)** Physical map of ABC1 in genomic DNA from *N. parvum* wild-type strain; **(B)** deletion mutant of *NpABC1* with the G418-resistance (KanMx).

The ABC1 gene was successfully amplified (Supplementary Figure S1), and the gDNA and cDNA sequences of the gene were compared. The ABC1 gene was found to be 1,773 bp in length and contained two exons (Figure 6). The gene was predicted to code for a 574 amino acid (aa) protein, consisting of two halves: one half is the ABC transporter transmembrane region, and the other half contains ATPase components. A P-loop motif was present, which is a characteristic feature of ABC transporters. ABC1 showed the highest similarity to a *Lasiodiplodia theobromae* (66.19%) ABC transporter protein (accession no. XP_035368823.1) and a heavy metal tolerance protein (accession no. KAB2580447.1).

#### Construction of Knockout and Complementation Plasmids

The upstream and downstream fragments of ABC1 gene and two fusion fragments were preliminarily determined by agarose gel electrophoresis (Supplementary Figures S2, S3), purified and recovered, and sequenced. It was found that all sequences were 100% similar to the predicted sequence. The positive clones were stored as the knockout plasmids.

The ABC1 gene fragment obtained from plasmid pClone007-ABC1 cDNA was detected by agarose gel electrophoresis (Supplementary Figure S4). The front and end of the gene fragment were introduced into the upstream and downstream homologous sequences of BHamI, the enzyme digestion site of vector pAN7-1, respectively. The results of enzyme digestion verification of recombinant plasmid pAN7-1-ABC1 were detected by agarose gel electrophoresis (Supplementary Figure S5). Positive clones verified by enzymatic digestion were stored as the complementation plasmids.

#### Preparation of Protoplasts and Genetic Transformation

**Figure 7:**
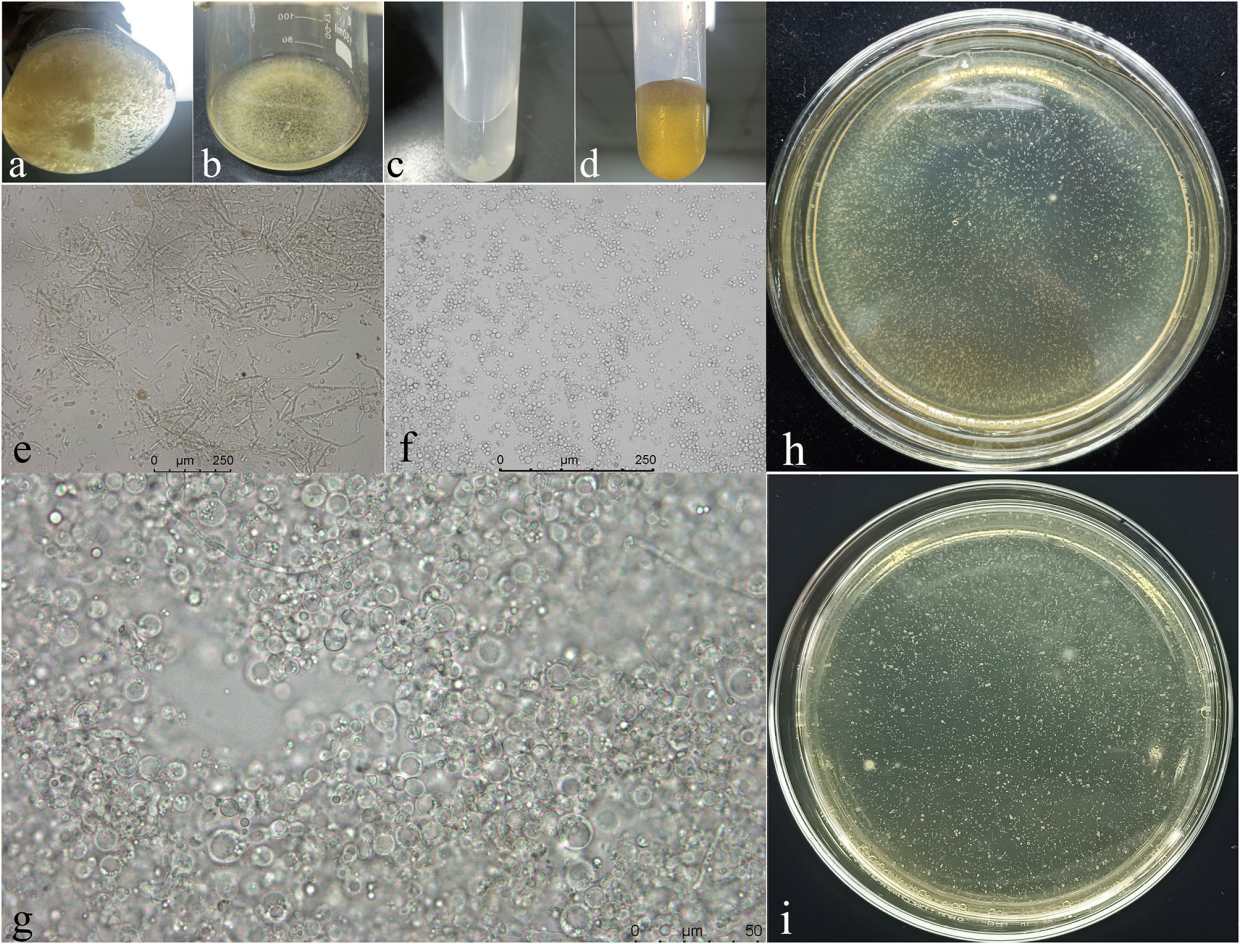
The process of mycelium cleavage into protoplasts and the recovery of protoplasts. **(a,e)**, mycelium enzymolysis for 3 h; **(b,f)**, mycelium enzymolysis for 8 h; **(c)**, protoplast precipitation after washing and centrifugation; **(d,g)**, protoplast recovery after genetic transformation; **(h,i)**, the colonies of transformants strains cultured at 25°C for 3 days.

The mycelium gradually collapsed and deformed in the process of enzymatic hydrolysis and finally obtained thin-walled, transparent, spherical protoplasts of different sizes. After genetic transformation and culture, the protoplasts were resuscitated and the cell wall in which it was regenerated could be visualized microscopically. After 3 days of culture on the plates, some tiny white single colonies can be observed, which are the transformant strains (Figure 7).

Deletion mutant strains (Δ*NpABC1*) and complemented strains (*NpABC1c)* were detected using the primers “KanMx,” “ABC1,” “Detection fragment 1,” “Detection fragment 2,” and “Hgy” (Table 2). The results were checked by agarose gel electrophoresis (Supplementary Figures S6, S7), and all the sequences results of positive strains were consistent with the predicted results.

#### Phenotypic Analysis of Transformants

**Figure 8:**
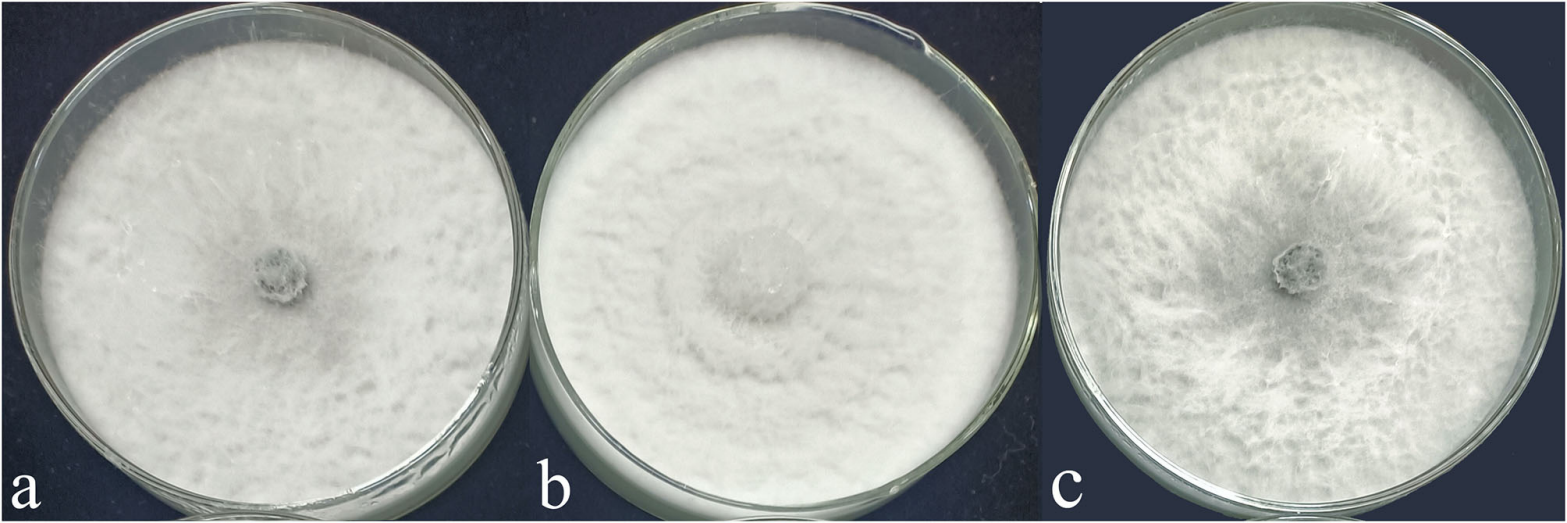
Colony of wild-type, deletion mutant and complementary strains of *N. parvum* cultured for 5 days. **(a)**, wild-type strain; **(b)**, deletion mutant strain; **(c)**, complementary strain; culture conditions: 25°C light–dark alternation for 12 h.

**Figure 9:**
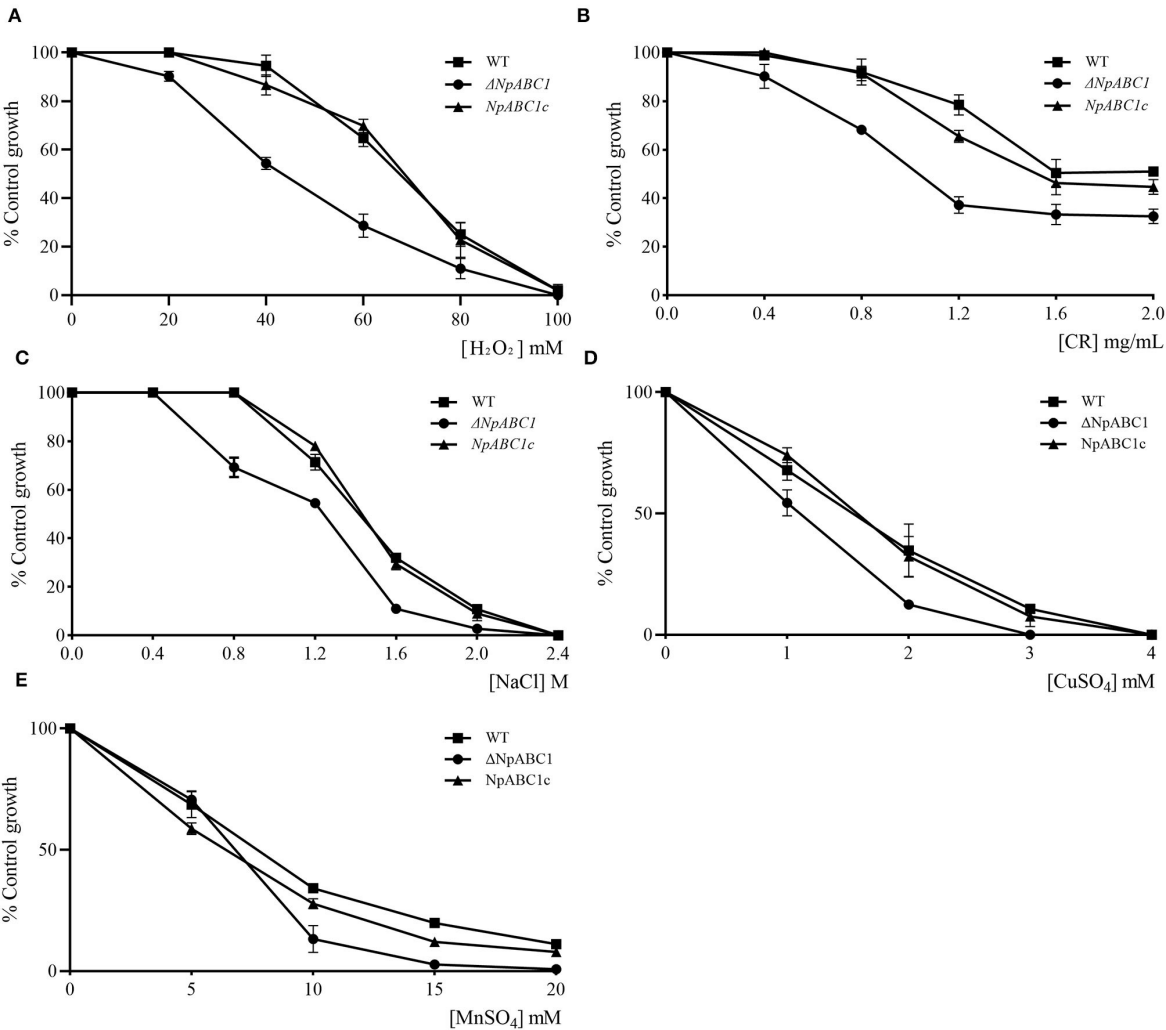
Effect of H_2_O_2_, CR, NaCl, and metal in *N. parvum* strains. **(A)** hydrogen peroxide, **(B)** Congo Red, **(C)** Sodium chloride, **(D)** copper, or **(E)** manganese. WT, wild-type strain (■); *ΔNpABC1*, deletion mutant strains (•); *NpABC1c*, complementary strains (▴). The X-axis represents the final concentration of the substance added to the PDA medium. The Y-axis (% control growth) represents the ratio of the colony diameter of the treatment group to the negative control group. Data of diameter were harvested after 3 days of growth. Culture conditions: 25°C light–dark alternation for 12 h.

The phenotypes of the mutant *ΔNpABC1* were different from the phenotypes of the wild-type and complementary strain *NpABC1c* (Figure 8). The aerial hyphae of the *ΔNpABC1* mutant were denser than those of the wild type, and the basal hyphae produced less pigment. However, there was no significant difference in the growth rate of these colonies. Based on the percent growth of the strains relative to controls, mutant *ΔNpABC1* was significantly more sensitive to the oxidative stress of H_2_O_2_ than WT and *NpABC1c* (Figure 9A); *ΔNpABC1* mutants also exhibited decreased tolerance to CR (Figure 9B). The hyperosmotic stress of NaCl caused significant differences in the colony growth of *ΔNpABC1* mutants (Figure 9C). These results suggest that ABC1 is required for vegetative growth and stress responses.

#### Drug Resistance and Metal Toxicity

The deletion mutant *ΔNpABC1* showed increased sensitivity to chloramphenicol, whereas the difference in growth was no significant in the case of carbendazim and tebuconazole (Table 4). The deletion mutant*ΔNpABC1* showed increased sensitivity to manganese (MnSO_4_) and copper (CuSO_4_) (Figures 9D,E).

**Table 4:** Drug sensitivity of *N. parvum* strains.

**Compound**	**WT EC_50_ (μg/mL)**	**WT MIC (μg/mL)**	**ΔNpABC1 EC_50_ (μg/mL)**	**ΔNpABC1 MIC (μg/mL)**	**NpABC1c EC_50_ (μg/mL)**	**NpABC1c MIC (μg/mL)**
Chloramphenicol	>100	>100	12.0	100	>100	>100
Carbendazim	0.005	0.6	0.001	0.8	0.006	0.6
Tebuconazole	0.05	2.5	0.06	2.5	0.05	2.5

#### Pathogenicity Test of Transformants

**Figure 10:**
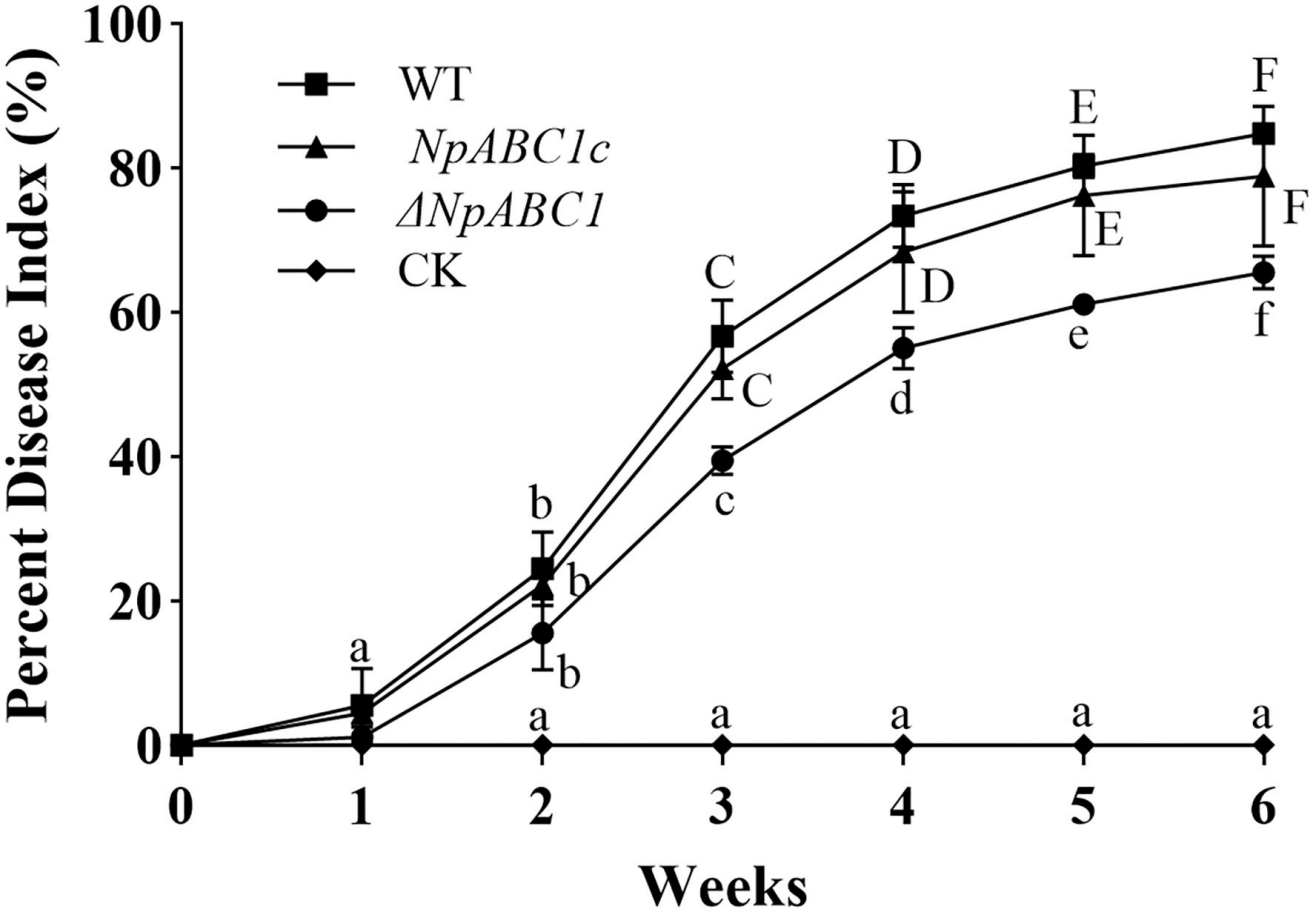
Dynamic changes in the disease index of walnut infected by different strains. WT, plants inoculated with wild-type strain (■); *ΔNpABC1*, plants inoculated with deletion mutant strain (•); *NpABC1c*, plants inoculated with complementary strain (▴); CK, negative control, plants inoculated with sterile water (♦). All assays were repeated three times, and the data were analyzed using one-way ANOVA and Duncan's range test in SPSS 16.0. Uppercases and lowercases indicate that the disease index of different treatment groups in the same period is significantly different (*p* ≤ 0.05).

**Figure 11:**
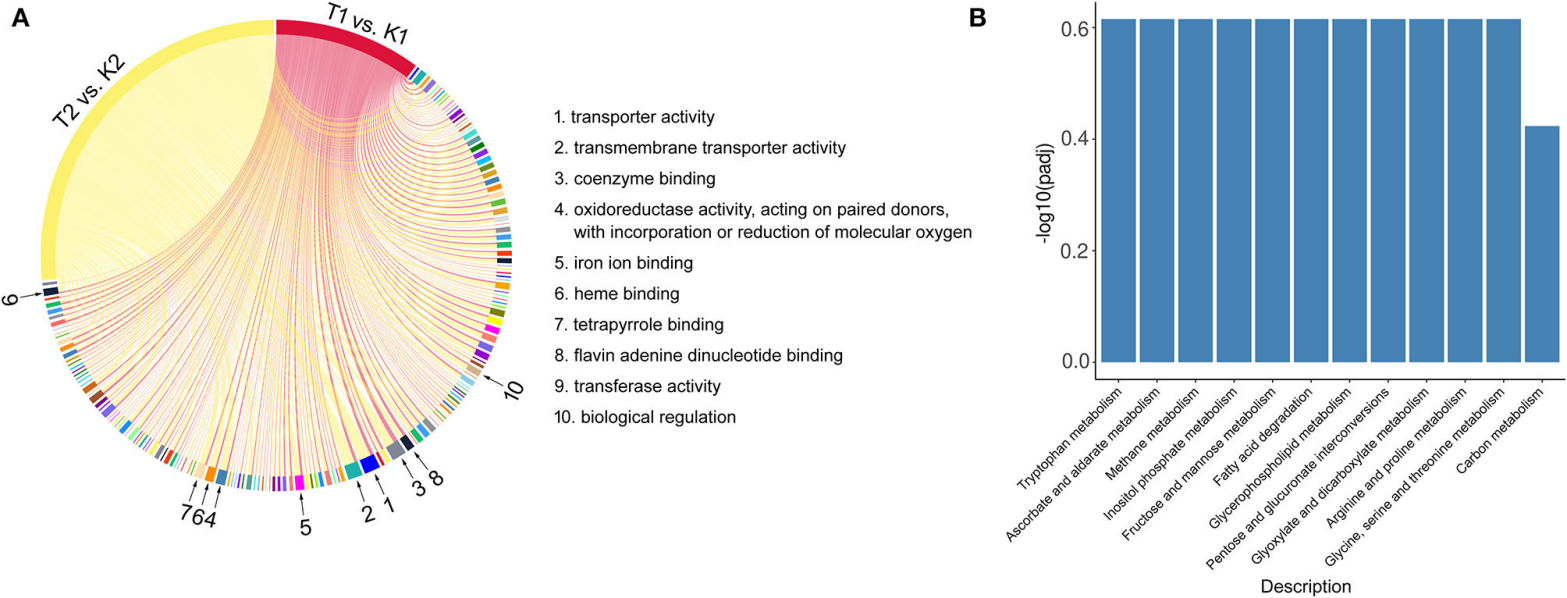
The common terms and pathways of GO and KEGG enrichment in T1 vs. K1 and T2 vs. K2 comparison groups. **(A)** Chord diagram of common terms in GO richment analysis. **(B)** Histogram of common pathways in KEGG richment analysis.

To determine whether *NpABC1* is involved in pathogenicity, we performed a pathogenicity test on branches by inoculating them with mycelial plugs of the wild-type, *ΔNpABC1* deletion mutant and complementary strains, and using the treatment group inoculated with pure PDA medium as a control (Figure 10). Mild symptoms were found on the *ΔNpABC1* mutant-infected twigs after culture (temperature: 20°C, humidity: 65%), whereas obvious symptoms were found for the wild-type and complementary strains. The statistical results of the disease index are shown in Figure 11. There was no significant difference in the disease index between the wild-type and complementary strains. However, the disease index caused by the *ΔNpABC1* mutant was significantly lower than that of the wild-type and complementary strains. This result suggests that *ABC*1 plays an important role in *N. parvum* virulence.

## Discussion

The results of transcriptome differential analysis illustrated that the wide range of differential gene enrichment of the three comparison groups shows that there are great differences in the gene expression patterns between the two strains and different conditions. Nevertheless, we found some commonalities. The comparison groups T1 vs. K1 and T2 vs. K2 have some significantly co-enriched GO terms and KEGG pathways, suggesting the feasibility of using transcriptome sequencing to study virulence factors of *N. parvum*. These co-term enriched in GO (Figure 11A), including transporter activity, transmembrane transporter activity, coenzyme binding, oxidoreductase activity, iron ion binding, heme binding, tetrapyrrole binding, flavin adenine dinucleotide binding, transferase activity, biological regulation, etc., for a total of 162, while the co-pathway enriched in KEGG (Figure 11B), including tryptophan metabolism, ascorbate and aldarate metabolism, methane metabolism, inositol phosphate metabolism, fructose and mannose metabolism, fatty acid degradation, glycerophospholipid metabolism, pentose and glucuronate interconversions, glyoxylate and dicarboxylate metabolism, arginine and proline metabolism, glycine, serine and threonine metabolism, and carbon metabolism, for a total of 12.

We found a number of intriguing DEGs in the three comparison groups simultaneously. For example, the genes are related to signal transduction, carbohydrate metabolism, response to peroxidative stress, biosynthesis of secondary metabolites, and transmembrane transport. Notably, histidine kinase (HK) and calcineurin (CaN) genes in signal transduction. The signal transduction of the interaction between the hosts and pathogens is very important during the infection. For pathogenic fungi, abundant signaling pathways are necessary for faster host recognition and successful colonization (Zhang et al., 2002; Catlett et al., 2003; Fujimura et al., 2003). The calmodulin signaling pathway and three well-conserved mitogen-activated protein kinase (MAPK) pathways play a critical role in this regard (Bahn, 2008; Zhang et al., 2021). Moreover, histidine kinase is a positive regulator of the Hog1 MAPK pathway (Yoshimi et al., 2005). Based on the differential expression of KH and CaN, we infer that the Hog1 MAPK and calcineurin signaling pathway play the important roles in the pathogenicity of *N. parvum*.

The upregulated differential genes about glycoside hydrolases (GHs), polysaccharide lyases (PLs), and carbohydrate esterases (CE) were significantly in carbohydrate metabolism. Many studies have shown that CWDEs, what the fungi produced during infection, are the important virulence factors of many plant pathogens (Blanco-Ulate et al., 2013; Rafiei et al., 2021). In addition, carbohydrates also can be released by degrading the cell wall to provide nutrients to pathogens (Morales-Cruz et al., 2015; Pérez-Hernández et al., 2017). On the other hand, plants can recognize pathogenic attack by detecting certain products to activate the plant immune response (Lagaert et al., 2009). Among them, GHs, PLs, and CE can promote lysis of the cell wall.

After breaking through the physical defenses of the host plant, the pathogen also needs to overcome the host's immune response. During the infestation, the rapid reactive oxygen species (ROS) burst of the host plant is one of the first defense responses (Mir et al., 2015). Therefore, the ability to process the ROS produced by the host is an important virulence factor of the pathogens (Zámocký et al., 2009; Guo et al., 2011). In addition, fungal resistance to H_2_O_2_ and its peroxidase activity are significantly and positively correlated with its pathogenicity, and peroxidase is a component of the antioxidant defense system (Mir et al., 2015). Therefore, we found that peroxidases are very dramatic in the annotation of DEGs related to oxidative stress response, which may imply that the strong pathogenicity of N. parvum to walnut is decided by its excellent ROS detoxification.

Annotation of upregulated differential genes belonging to biosynthesis of secondary metabolites heavily identified some key genes related to toxin synthesis proteins. As we all know, toxins are the important weapons of pathogenic fungi. Toxins may disable host cellular functions or kill host cells before infection, or during a necrotrophic stage of infection (Idnurm and Howlett, 2001; Tsuge et al., 2013). Toxins are generally the products of the secondary metabolism of fungi. Most of these products are structurally heterogeneous small molecules, which are mainly derived from polyketide compounds or nonribosomal peptides. They can harm host cells by interfering with enzymatic reactions and cell transport or by destroying the cell membrane (Andolfi et al., 2011), while a variety of fungal toxins have been studied in fungi, such as ToxA (Ciuffetti et al., 2010), HC-toxin (Brosch et al., 1995), AM-toxin (Johnson et al., 2000), AAL-toxin (Akamatsu et al., 1997), and AK-toxin (Tanaka and Tsuge, 2000), but many toxins and their mechanisms of action are waiting to be discovered.

The upregulated differential genes of ABC transporters were significantly in transmembrane transport. In this study, we observed that the symptoms of plant disease were different between wild-type and mutant strains by knocking out ABC1. At the same time, a knockout complementation experiment excluded epigenetic changes caused by the mutagenicity of protoplast transformation itself. This result indicates that the virulence of the mutant strain *ΔNpABC1* was lower than that of wild-type *N. parvum* to a certain extent. Previous work indicates that ABC is responsible for virulence. ABC in *Fusarium graminearum* plays an important and diverse role in drug resistance and pathogenesis (Abou Ammar et al., 2013). Our results are also consistent with the conclusion, the abc4 gene in *Magnaporthe oryzae* has an important impact on virulence, and mutant abc4 cannot infect intact leaves and causes less intense diseases on damaged leaves (Gupta and Chattoo Bharat, 2007). As we show that deletion of the ABC1 in *N. parvum* causes low-intensity disease on walnuts. At the same time, the ABC plays a role in the protection species against oxidative stress that has been indicated, highly expressed in response to various stresses (Wolfger et al., 2001; Moons, 2003; Lynch et al., 2009; Bogomolnaya et al., 2013). Our data show that the mutant strain *ΔNpABC1* was sensitive to H_2_O_2_. So, we infer that the ABC1 may also play an important role in response peroxide stress in the walnut for *N. parvum*. In addition, the deletion of the *NpABC1* gene led to the increased sensitivity of the strain to cell wall disturbance stress (exposure to CR) and high osmotic pressure (exposure to NaCl), suggesting that ABC1 may be involved in cell wall synthesis (Martin et al., 2021) and protecting cells from osmotic stress (Van der Heide and Poolman, 2000; Van der Heide, 2001; Nagayama et al., 2014). ABC transporters are often thought to increase resistance, and the most famous of the members are multidrug resistance proteins (MDRs), multidrug resistance-associated proteins (MRPs), and pleiotropic drug resistance (PDR) protein subfamilies (Wolfger et al., 2001). Therefore, we conducted sensitivity experiments on some common drugs. The deletion mutant *ΔNpABC1* showed increased sensitivity to chloramphenicol but had no significant change in sensitivity to carbendazim and tebuconazole, indicating that the ABC1 gene may not be involved in multidrug resistance but in antibiotic. Besides, since the members of ABC subfamily have vacuolar detoxification (such as the MRPs), which leads them to develop heavy metal resistance (Wemmie et al., 1994; Li et al., 1997; Bauer et al., 1999). It is consistent with the results of our strains metal sensitivity experiments in which the mutant strains *ΔNpABC1* showed a significant increase in sensitivity to Mn2^+^ and Cu^2+^. In summary, our data indicate that ABC1 plays an important role in the pathogenesis of *N. parvum* because the ABC1 gene helps the pathogens to cope with the adverse environment in the process of pathogen–host interactions.

## Data Availability Statement

Publicly available datasets were analyzed in this study. This data can be found here: https://www.ncbi.nlm.nih.gov/sra/?term=Transcriptome+sequencing+of+Neofusicoccum+parvum. All transcriptomic data of the 12 samples of *N. parvum* were deposited in the NCBI Sequence Reads Archive (SRA) under the accession number SRX11046476-11046487.

## Author Contributions

Conceptualization, methodology, and writing—original draft preparation: JC. Software: JC and MW. Validation: TZ and SH. Formal analysis: JC and SH. Investigation: TQ and SL. Resources: MW and SL. Data curation: TQ and SH. Writing, reviewing, and editing: SL and TL. Visualization and supervision: SH. Project administration: TZ. Funding acquisition: HZ. All authors contributed to the article and approved the submitted version.

## Funding

This research was funded by Science and Technology Department of Sichuan Province, grant number 22YYJC3079.

## Conflict of Interest

The authors declare that the research was conducted in the absence of any commercial or financial relationships that could be construed as a potential conflict of interest.

## Publisher's Note

All claims expressed in this article are solely those of the authors and do not necessarily represent those of their affiliated organizations, or those of the publisher, the editors and the reviewers. Any product that may be evaluated in this article, or claim that may be made by its manufacturer, is not guaranteed or endorsed by the publisher.
