# Supplementary material for: Comparative Transcriptomics and Gene Knockout Reveal Virulence Factors of Neofusicoccum parvum in Walnut
Source: Front Microbiol. 2022 Jul 15;13:926620. doi: 10.3389/fmicb.2022.926620 (PMC9335079; doi:10.3389/fmicb.2022.926620)
Supplement: Supplementary file 1 [file Data_Sheet_1.pdf]

Supplementary Material

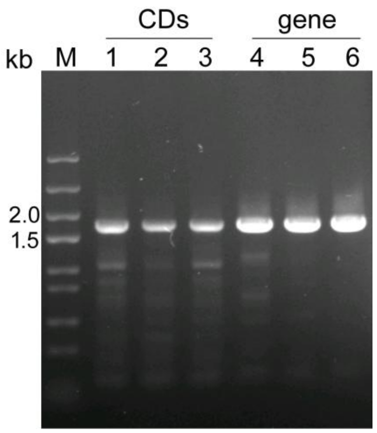

Supplementary Figure 1. ABC1 gene fragments were amplified from genome and cDNA by PCR

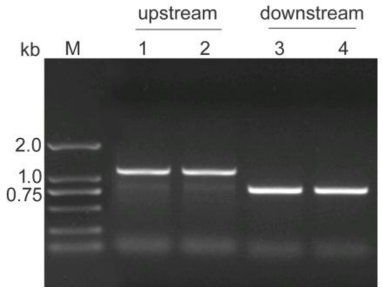

Supplementary Figure 2. The upstream and downstream homologous arm of ABC1 gene was amplified from the genome by PCR

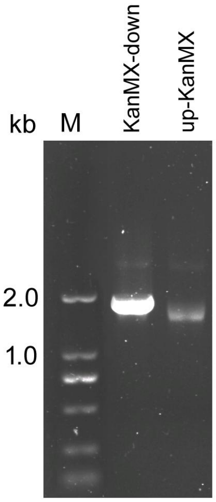

Supplementary Figure 3. The fusion fragment of target gene KanMX and homologous arm was obtained by PCR

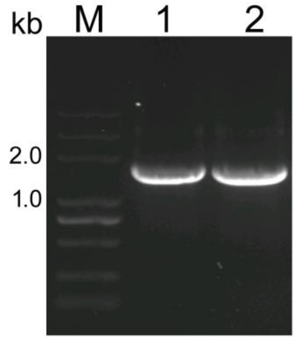

Supplementary Figure 4. The ABC1 gene fragment in cDNA was added with the homologous linker of vector pan7-1 by PCR

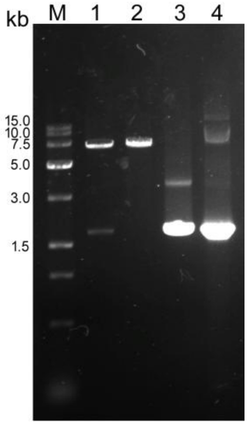

Supplementary Figure 5. Detection of complementation plasmids

Note: 1,The recombinant plasmid was digested by restriction enzyme BHamI; 2, Linearized plasmid; 3, ABC1; 4, ABC1 fragment was amplified from recombinant plasmid by PCR

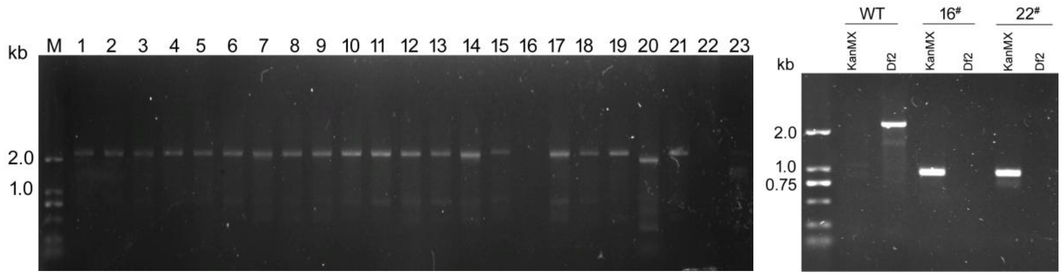

Supplementary Figure 6. Detection of mutant strain ΔNpABC1

Note: Detection of fragment 1 (left);Detection of fragment 2 and KanMX (right)

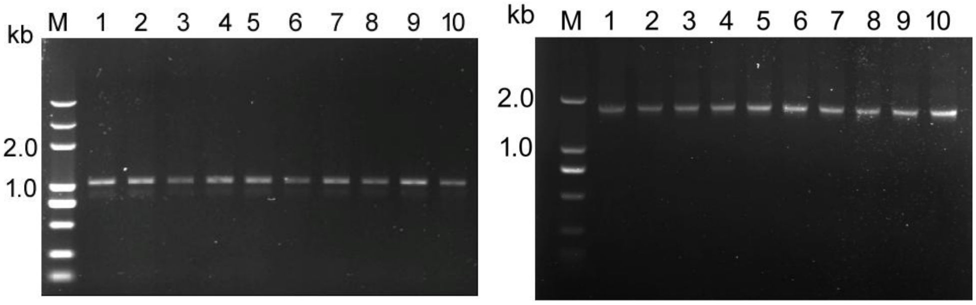

Supplementary Figure 7. Detection of complementary strain *NpABC1c*

Note: Detection of Hgy (left);Detection of fragment ABC1 (right)

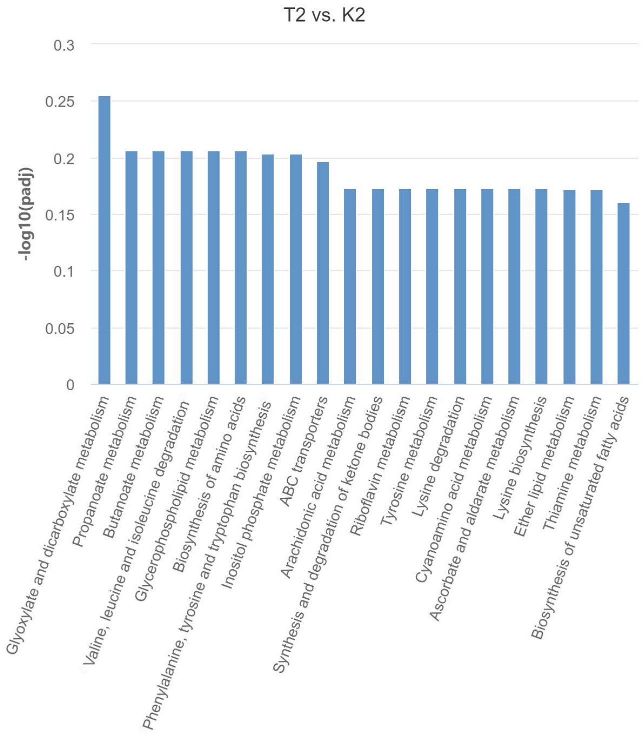

Supplementary Figure 8. KEGG pathway of comparison group T2 vs. K2 (top item 21-40)
